# Rapid molecular species identification of mammalian scat samples using nanopore adaptive sampling

**DOI:** 10.1093/jmammal/gyae044

**Published:** 2024-05-06

**Authors:** Lexi E Frank, Laramie L Lindsey, Evan J Kipp, Christopher Faulk, Suzanne Stone, Tanya M Roerick, Seth A Moore, Tiffany M Wolf, Peter A Larsen

**Affiliations:** Department of Veterinary and Biomedical Sciences, University of Minnesota, St. Paul, MN 55108, United States; Department of Veterinary and Biomedical Sciences, University of Minnesota, St. Paul, MN 55108, United States; Department of Veterinary and Biomedical Sciences, University of Minnesota, St. Paul, MN 55108, United States; Department of Animal Science, University of Minnesota, St. Paul, MN 55108, United States; Department of Veterinary and Biomedical Sciences, University of Minnesota, St. Paul, MN 55108, United States; Leech Lake Band of Ojibwe, Cass Lake, MN 56633, United States; Grand Portage Band of Lake Superior Chippewa, Grand Portage, MN 55605, United States; Department of Veterinary Population Medicine, University of Minnesota, St. Paul, MN 55108, United States; Department of Veterinary and Biomedical Sciences, University of Minnesota, St. Paul, MN 55108, United States

**Keywords:** fecal DNA, MinION, mitochondrial DNA, molecular barcoding, nanopore sequencing, phylogenetics, species identification

## Abstract

Accurate taxonomic species identification is essential to the study of mammals. Despite this necessity, rapid and accurate identification of cryptic, understudied, and elusive mammals remains challenging. Traditional barcoding of mitochondrial genes is standard for molecular identification but requires time-consuming wet-lab methodologies. Recent bioinformatic advancements for nanopore sequencing data offer exciting opportunities for noninvasive and field-based identification of mammals. Nanopore adaptive sampling (NAS), a polymerase chain reaction (PCR)-free method, selectively sequences regions of DNA according to user-specified reference databases. Here, we utilized NAS to enrich mammalian mitochondrial genome sequencing to identify species. Fecal DNA extractions were sequenced from 9 mammals, several collected in collaboration with Minnesota Tribal Nations, to demonstrate utility for NAS barcoding of noninvasive samples. By mapping to the entire National Center for Biotechnology Information mammalian mitochondrial reference genome database and bioinformatically analyzing highly similar matches, we successfully produced species identifications for all fecal samples. Eight of 9 species identifications matched previous PCR or animal/fecal appearance-based identifications. For the ninth species, our genetic data indicate a misidentification stemming from the original study. Our approach has a range of applications—particularly in field-based wildlife research, conservation, disease surveillance, and monitoring of wildlife trade. Of importance to Minnesota tribes is invasive species monitoring, detections, and confirmation as climate impacts cause changes in biodiversity and shifts in species distributions. The rapid assessment techniques described here will be useful as new introductions and range expansions of native and invasive species may first be detected by the presence of signs such as scat rather than direct observations and will be helpful for chronically understaffed tribal natural resources agencies.

## Teaser

Taxonomic species identification of mammals is essential in mammalogy. We utilized nanopore adaptive sampling, a PCR-free method, to enrich mitochondrial genome sequencing according to a user-specified database to bioinformatically identify 9 species from fecal DNA of mammals.

Taxonomic species identifications are essential to the study of mammals, especially for ecological, biodiversity, and conservation-based studies. Nevertheless, an accurate identification can be difficult, especially between cryptic, elusive, and endangered species that are rare to observe in nature or within natural history collections (Baker and Bradley 2006). An array of methods, with varying levels of accuracy, are routinely used to identify mammalian species ranging from direct field observation and examination of external morphological characteristics to morphometric analyses of subtle cranial features and molecular barcoding using DNA (De Barba et al. 2014; Walker et al. 2016; Hedrick and Dumont 2018; Potter et al. 2019). Of these methods, molecular approaches that facilitate the phylogenetic, phylogeographic, and molecular systematic analyses of mammals have led to a large increase of the recognized mammalian species in nature, especially among taxa of bats, rodents, and shrews (Bradley and Baker 2001; Redondo et al. 2008; Baird et al. 2015; Giarla and Esselstyn 2015; D’Elía et al. 2019; DeSalle and Goldstein 2019).

Advancements in molecular technologies and associated methodologies continually provide new research opportunities for the study and identification of mammals (Larsen and Matocq 2019). For example, DNA can be extracted from fecal samples and used for host species identification (Höss et al. 1992). Feces is an easy-to-acquire, noninvasive sample that can be used to elucidate many biological features of the depositing host species (e.g., genetics, diet, metacommunities) without needing to handle or visualize the animal directly (Kohn and Wayne 1997; Valentini et al. 2009; Srivathsan et al. 2016; Kusack et al. 2022; Pannoni et al. 2022). Although visual examination of mammalian feces—including coloring, size, and shape—is routinely used to distinguish species, even subject matter experts can fail to produce an accurate species identification from physical characteristics of feces alone (Davison et al. 2002). More recently, molecular techniques using fecal DNA have proven useful to achieve more accurate identifications. Sloughed rectal cells from the excreting individual are present in the feces and can be used to extract host-specific DNA (Höss et al. 1992). An early and still widely used approach consists of polymerase chain reaction (PCR) amplification of host barcoding genes, typically the mitochondrial cytochrome-*b* (*Cytb*) and/or cytochrome oxidase I (*COI*) gene for mammals, and sequencing of fecal DNA, with a wide variety of downstream applications ranging from phylogenetics to forensics (Höss et al. 1992; Palomares et al. 2002; Dalén et al. 2004). For example, this method was used to investigate the genetics of a threatened bear population in Europe that consisted of less than 10 individuals spread across a large geographic area (Höss et al. 1992). In another study, species-specific primers were used to identify feces from the elusive and rare *Lynx pardinus* (Iberian Lynx); however, low concentrations and quality of DNA generated false negatives (Palomares et al. 2002). In a similar study, researchers identified species from fecal samples of multiple sympatric carnivores by utilizing a multiplexed PCR system that produced fragments of different lengths for each species (Dalén et al. 2004). However, this method could only account for species included in the specific set of primers targeted for the study and was challenged by false negatives (Dalén et al. 2004).

Collectively, the utility of PCR and universal or specific primers for the identification of mammal species from scat suffers from several limitations (Franco-Sierra and Díaz-Nieto 2020). Despite the ease of collection, fecal samples are composed of a complex mix of biological material—including bacteria—that can inhibit downstream molecular techniques, especially PCR (Kohn et al. 1995). However, various inhibition buffers including BSA can be used to overcome PCR inhibitors (Schrader et al. 2012). The presence of bacteria, the initial warm and damp environment of feces, and subsequent environmental exposure leads to degradation of host DNA (Kohn et al. 1995). Moreover, current PCR-based molecular techniques for scat-based identification largely require molecular-grade laboratory conditions and robust equipment, meaning that time to identification can be substantial and the techniques are typically not performed in the field (Erlich 2015). There are also cases where PCR is not a viable option due to factors including primer availability and standardization of reactions (Franco-Sierra and Díaz-Nieto 2020).

Other methods for species identification with noninvasive samples include high-resolution melting analysis, which can be effective for determining species (Buglione et al. 2020). However, this method still requires PCR, nonportable equipment, and the species-specific melting profiles of positive controls (Buglione et al. 2020). Another method called FecalSeq uses differences in vertebrate and bacterial methylation to capture excreting host DNA from fecal samples that can then be used for downstream sequencing (e.g., shotgun and reduced representation sequencing; Chiou and Bergey 2018). While this method is highly effective at enriching vertebrate DNA, it cannot distinguish between groups of vertebrates and adds 24 h of technician time to the workflow (Chiou and Bergey 2018).

Genomic technologies have advanced rapidly over the past few decades and are impacting the field of mammalogy in remarkable ways (Larsen and Matocq 2019). One of the most exciting advancements pertains to single-molecule nanopore sequencing, with a variety of sequencing platforms and applications introduced by Oxford Nanopore Technologies (ONT; Oxford Nanopore Technologies 2023). The relative ease of setup and low capital costs of the sequencer (e.g., MinION) make this technology more accessible compared to traditional sequencing platforms (Laver et al. 2015; Franco-Sierra and Díaz-Nieto 2020). Since 2014, the per-base accuracy of ONT sequencing platforms has steadily improved, with current raw sequence rates achieving greater than 99% accuracy per base (Oxford Nanopore Technologies 2023). When combined with the field-deployable ONT MinION sequencer, such improvements open the door to a wide variety of applications for field-based molecular research. In parallel to ONT hardware and sequencing chemistry improvements, significant advancements have been made in bioinformatic algorithms for the real-time analyses of ONT sequence data (Oxford Nanopore Technologies 2023). In particular, the recently released nanopore adaptive sampling (NAS) software (i.e., ReadUntil) can be used to selectively sequence individual molecules of DNA, cDNA, or RNA (Payne et al. 2021; Martin et al. 2022; Kipp et al. 2023). The major bottleneck of next-generation sequencing, such as nanopore sequencing, is bioinformatic analysis and interpretation of results, highlighting the importance of the creation of and public accessibility to workflows that analyze this abundance of data (Sboner et al. 2011).

**Fig. 1.**
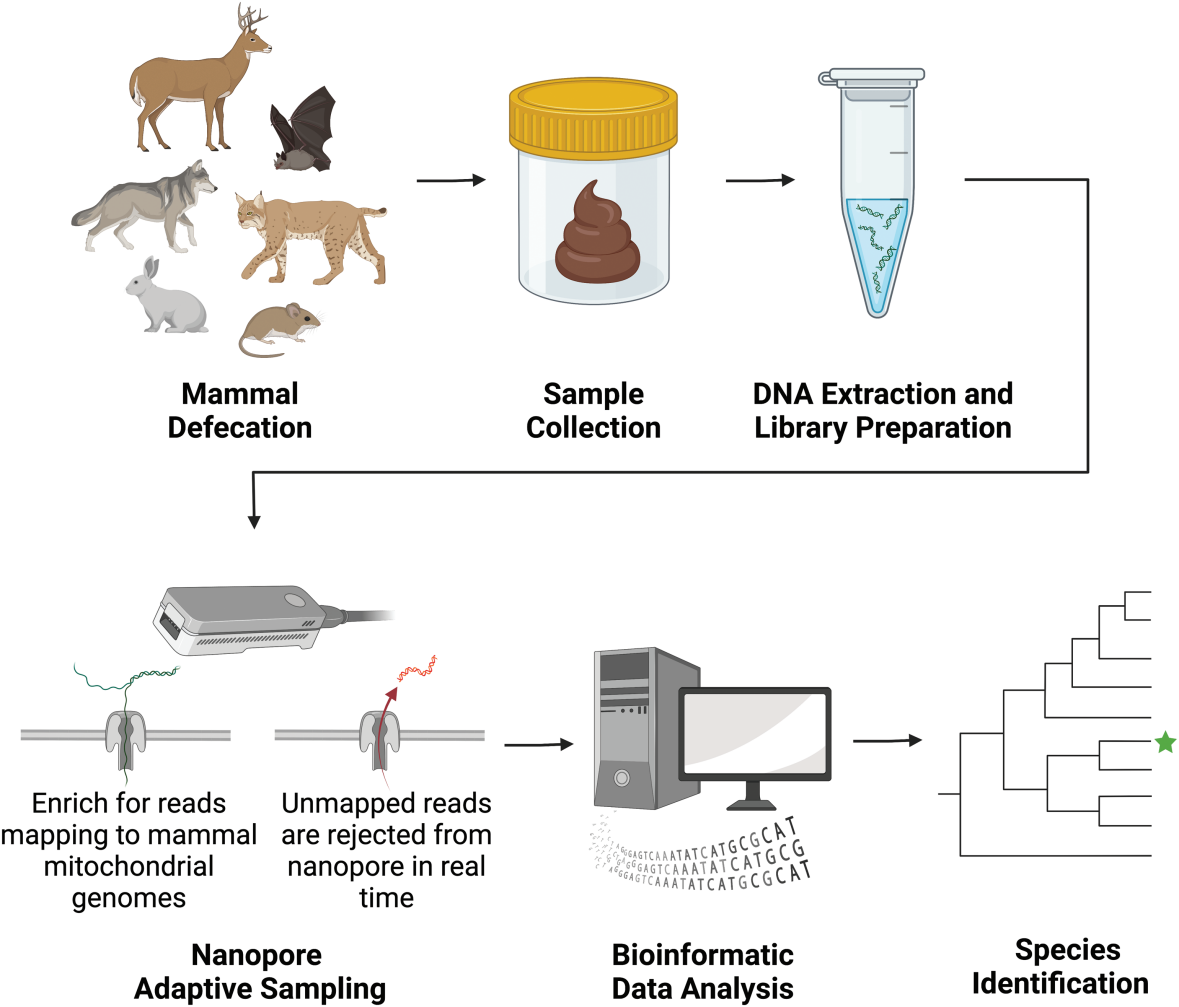
Species identification workflow using NAS. Mammal feces is collected from the environment and whole genomic DNA is extracted. DNA extracts are prepared for Oxford Nanopore Sequencing following protocols for genomic DNA (see Materials and methods). NAS enriches for sequencing of mammalian mitochondrial genomes. Sequenced reads are bioinformatically analyzed to determine species. Figure created with BioRender.com.

As individual molecules are being sequenced within a given nanopore, NAS utilizes an advanced bioinformatic workflow (e.g., minimap2) to compare the resulting nucleotides to a user-specified reference file (e.g., all publicly available mammalian mitochondrial genomes), with real-time results generated during a given sequencing experiment (Payne et al. 2021). Approximately every 0.4 s, a sequence of a given molecule is compared to the reference (Payne et al. 2021). Any sequences having a minimum of ~70% similarity to the reference database will be retained and those that are below ~70% similarity to the database are rejected (Payne et al. 2021). Therefore, targets of interest can be selectively enriched (e.g., mitochondrial DNA, specific genes, pathogen genomes, etc.) and nontarget DNA is rejected from the sequencing pore. NAS can effectively be applied for host species identification of fecal samples by rejecting nonmammalian DNA and enriching for putative host DNA (Fig. 1; Payne et al. 2021; Wanner et al. 2021). In particular, the method is ideally suited for the targeted enrichment of mammalian mitochondrial DNA, present in high copy numbers in cells which helps avoid obstacles associated with degraded samples or highly repetitive DNA (Wanner et al. 2021). An important aspect of NAS is that the length of a sequenced read is not restricted to template length and single-molecule sequences can be thousands of bases long (Oxford Nanopore Technologies 2023). Long reads with overlapping sequences of DNA can be used to increase confidence of downstream taxonomic identifications and be used to assemble entire mitochondrial genomes. Importantly, this methodology does not require PCR to amplify specific barcoding genes (e.g., *Cytb* or *COI*).

Indigenous peoples have long been stewards of biodiversity globally and have interest in maintaining strong stewardship principles using both traditional and modern techniques (Fletcher et al. 2021). Practical applications of rapid species identification technologies will assist tribal and other governmental entities in determination of mammalian species assemblages using nonintrusive techniques such as scat collections and opportunistic field collections. Our aim was to develop a workflow to enhance applied field assessments of biodiversity and invasive species monitoring using noninvasive fecal samples. In this study, we merged modern technological developments in genetic assessments with tribally collected data to evaluate the efficacy of rapid species detection capabilities.

Here we show how the NAS method can be leveraged for the rapid sequence-based molecular identification of a variety of mammalian species using DNA extracted from scat samples. This method is purely a bioinformatic approach that can be used for real-time mammalian species identification without PCR. Of particular interest to the research community is that the method can be performed with field-deployable equipment using a straightforward whole genomic DNA extraction and sequencing approach with mapping results reported during the analysis, thus facilitating rapid putative species identifications in both lab and field settings.

## Materials and methods

### Sample collection and DNA extraction

All 9 samples were collected in Minnesota between 2018 and 2022. The general locality of collection for all fecal samples examined herein is provided in Table 1. Of these, 5 were included in a previous study (Bernstein et al. 2021). These samples were collected at 2 sites from Minnesota Ojibwe tribes at the Leech Lake Reservation and Grand Portage Reservation (Table 1). For the 5 samples originating from Bernstein et al. (2021; Table 1), species identification was originally confirmed in 2 ways: (1) the sample was taken directly from an individual of a known species; or (2) DNA was extracted from the fecal samples followed by a PCR of the mitochondrial control region and visual scoring of the resulting amplicons (Bernstein et al. 2021). The remaining samples were collected from various sites in Minnesota (Table 1), including opportunistically from the Minnesota zoo and from captive animals. Samples were identified either by physical characteristics of the feces or by visual confirmation of the excreting mammal at the time of collection (Table 1). These species identifications were blinded for the NAS analysis and revealed for comparison after NAS-guided species identification was completed. Samples were collected in tubes or whirl-paks and stored at −80 °C until DNA extraction was performed. DNA was extracted from 9 samples—5 extractions were completed for the Bernstein et al. (2021) study and 4 were done specifically for the study herein—using the QIAamp PowerFecal Pro DNA Kit (QIAGEN, Hilden, Germany). DNA extracts were quantified using a Qubit 4 fluorometer (Invitrogen, Carlsbad, California). From each sample, the input DNA concentration used for library preparation ranged between 3.74 and 780 ng/μL. All newly collected fecal samples for the research conducted herein were opportunistically secured and did not require handling of individuals.

**Table 1.** NAS and mitochondrial mapping results from fecal samples leads to species identification. Field-based fecal IDs are based on visual examination and diameter of the feces during sample collection. NAS species IDs were generated using the workflow presented herein.

Sample ID	Collection location	Field-based fecal ID	Original species ID	Original ID confirmation method	NAS species ID
1^a^	Grand Portage, MN	—	*Canis lupus familiaris*	Physical characteristics of animal	*Canis lupus*
2^a^	Grand Portage, MN	*Canis lupus*	*Canis lupus*	PCR amplicon size	*Canis lupus*
3^a^	Leech Lake, MN	*Canis lupus*	*Vulpes vulpes*	PCR amplicon size	*Canis lupus*
4^a^	Leech Lake, MN	*Vulpes vulpes*	*Pekania pennanti*	PCR amplicon size	*Pekania pennanti*
5^a^	Leech Lake, MN	*Canis lupus*	*Lynx rufus*	PCR amplicon size	*Lynx rufus*
6	Saint Paul, MN	*Sylvilagus floridanus*	*Sylvilagus floridanus*	Physical characteristics of animal	*Sylvilagus floridanus*
7	Saint Paul, MN	Rodent spp.	Rodent spp.	Physical characteristics of animal	*Peromyscus leucopus*
8	Olmsted County, MN	—	*Odocoileus virginianus*	Physical characteristics of animal	*Odocoileus virginianus*
9	Minnesota Zoo, Apple Valley, MN	—	*Eidolon helvum*	Physical characteristics of animal	*Eidolon helvum*

^a^Indicates sample from Bernstein et al. (2021; see this study for more information on field-based ID and original species ID). Samples with no field-based fecal ID were collected from captive animals (Sample 8 from a captive white-tailed deer herd and Sample 9 from the zoo).

### Nanopore library preparation

Library preparation was performed blinded to sample species identification. Nine individual DNA libraries were constructed using the ONT Sequencing Ligation Kit SQKLSK109 or SQKLSK110 following manufacturer instructions for either the R9.4.1 Flongle (up to 126 sequencing nanopores, low throughput) or R9.4.1 full-sized MinION flow cell (up to 512 sequencing pores, high throughput) protocols. Total input DNA into library preparation from each sample ranged between approximately 500 and 1,000 ng, for use with the Flongle or full flow cell, respectively. To prepare the ends of DNA molecules in each sample for adapter attachment, we used NEBNext FFPE DNA Repair and Ultra II End Repair/dA-tailing Module reagents (New England Biolabs Inc., Ipswich, Massachusetts) and incubated at 20 °C for 5 min and then at 65 °C for 5 min. A 1:1 bead to sample ratio of AMPure XP beads (Beckman Coulter, Indianapolis, Indiana) was used for bead cleanup on a magnetic separation rack, following the ONT ligation kit protocol. For adapter ligation, Adapter Mix F (Oxford Nanopore Technologies, Oxford, United Kingdom), Ligation buffer (Oxford Nanopore Technologies, Oxford, United Kingdom), and NEBNext Quick T4 DNA Ligase (New England Biolabs Inc., Ipswich, Massachusetts) were added to the DNA sample from the previous steps and incubated for 10 min. This was followed by another bead cleanup (2:5 bead to sample ratio, as outlined by the ONT Ligation kit protocol) and addition of the ONT short fragment buffer to purify fragments of all sizes equally. The final library was eluted into elution buffer (Oxford Nanopore Technologies, Oxford, United Kingdom) of volume of 7 µL for Flongle sequencing or 15 µL for sequencing on a full flow cell at 37 °C and quantified by a Qubit 4 Fluorometer (Invitrogen, Carlsbad, California). Each library was stored at 4 °C before sequencing.

### Sequencing and basecalling

Each library was individually sequenced on a MinION with either a R9.4.1 Flongle flow cell or R9.4.1 full flow cell. The sequencing was performed on either a Linux desktop computer (Intel C600/X79 series i9-10920X 12 core; Linux 5.4.0-77-generic x86_64; Ubuntu 18.04; Nvidia Quadro RTX 4000 GPU with 8-GB video memory) or a Linux laptop (16x 11th gen Intel Core i7; Ubuntu 18.04; Nvidia GeForce RTX 3080 Ti GPU with 16-GB video memory). Flush tether and flush buffer (ONT) were mixed and loaded to prime the flow cell. The library, sequencing buffer II, and loading beads II (ONT) were combined and loaded into the flow cell. Sequencing parameters were set within the MinKNOW GUI (ONT, v4.3.20) with the adaptive sampling option turned on. The adaptive sampling reference file was created by gathering all reference mammal mitochondrial genomes available from National Center for Biotechnology Information (NCBI) and including them in a single reference file in FASTA format. This file includes all complete reference mitochondrial genome sequences from all mammal species available on NCBI at the time of file creation (14 March 2022). This file is selected during the setup of an adaptive sampling experiment to determine enrichment of reads and alignment in real time. Fast basecalling was chosen for real-time alignments to the reference file. Sequencing was initiated and run for 48 h or until the Flongle/flow cell was exhausted of pores. Raw FAST5 files generated during sequencing were basecalled post hoc with super accuracy by ONT Guppy basecaller (v5.0.11). Raw FASTQ nanopore sequence data from the experiments are deposited in the NCBI Sequencing Read Archive (SRA), under BioProject number PRJNA1026962.

### Bioinformatic processing

Detailed instructions describing the workflow used here are available in Supplementary Data SD1. Bioinformatic analysis was completed with access to the Minnesota Supercomputing Institute, which provided computational resources and data storage. For each sequencing run, metadata were generated using the NanoPlot (v1.32.1) and fastqc (v0.11.7) software packages (Babraham Bioinformatics 2010; De Coster and Rademakers 2023). FASTQ files generated for each sample were concatenated and then quality filtered using a score of 7 or higher and a read length between 300 bases and 17 kilobases using NanoFilt (v2.6.0; De Coster et al. 2018). This FASTQ was then aligned to the reference file containing all mammal mitochondrial genomes using minimap2 (v2.17; Li 2018). Files were indexed and organized using SAMtools (v1.9; Danecek et al. 2021). The filtered FASTQ for each sample with reads mapping to the mitochondrial database was then used as input into Kraken 2 (v2.1.2) to further filter the data for mammalian mitochondrial reads (Wood et al. 2019). Kraken 2 maps the reads to a reference database using a *k*-mer-based approach to provide taxonomic classifications of sequences. Here, the Kraken 2 reference database was created using the NCBI mitochondrial genome refseq file (O’Leary et al. 2016). Kraken 2 output files were used to visualize the data in Pavian software on R studio (v4.2.2; Breitwieser and Salzberg 2020). De novo assembly of the data was completed with Flye (v2.9.1), when possible, and contigs were used for phylogenetic analyses (Kolmogorov et al. 2019). Flye assembly contigs of near-complete mitochondrial genomes were annotated with the web-based annotator, Mitos2 (Donath et al. 2019).

### Phylogenetic analysis

When possible, barcoding genes (*Cytb* and *COI*) or any substantial and continuous sections of the mitochondrial genome were extracted from sequenced reads by aligning to matching reference mitochondrial genomes using Geneious Prime software (v2022.2.1). For rapid putative results, these barcoding genes or large sections of mitochondrial sequences were input into the NCBI Basic Local Alignment Search Tool (Blast) search engine and/or the Barcode of Life Data System (BOLD) identification search engine for *COI* genes (Altschul et al. 1990; Ratnasingham and Hebert 2007). These web-based search engines provide the top matching sequences from their databases with percent similarity calculations. The Blast search provides “Distance tree results” consisting of a neighbor-joining phylogenetic tree of our sample sequence with the matches generated by Blast. The BOLD search engine provides a “taxon ID tree” that generates a neighbor-joining tree based on our sample and their matching nucleotide sequences.

To confirm the rapid results provided by Blast and BOLD search engines, barcoding genes from the same species and closely related species, as well as an outgroup species, were collected from NCBI (accession numbers provided in figures). Phylogenetic trees were constructed using RaxML (v8.2.12) GAMMA model with 1,000 bootstrap iterations (Stamatakis 2014).

## Results

**Fig. 2.**
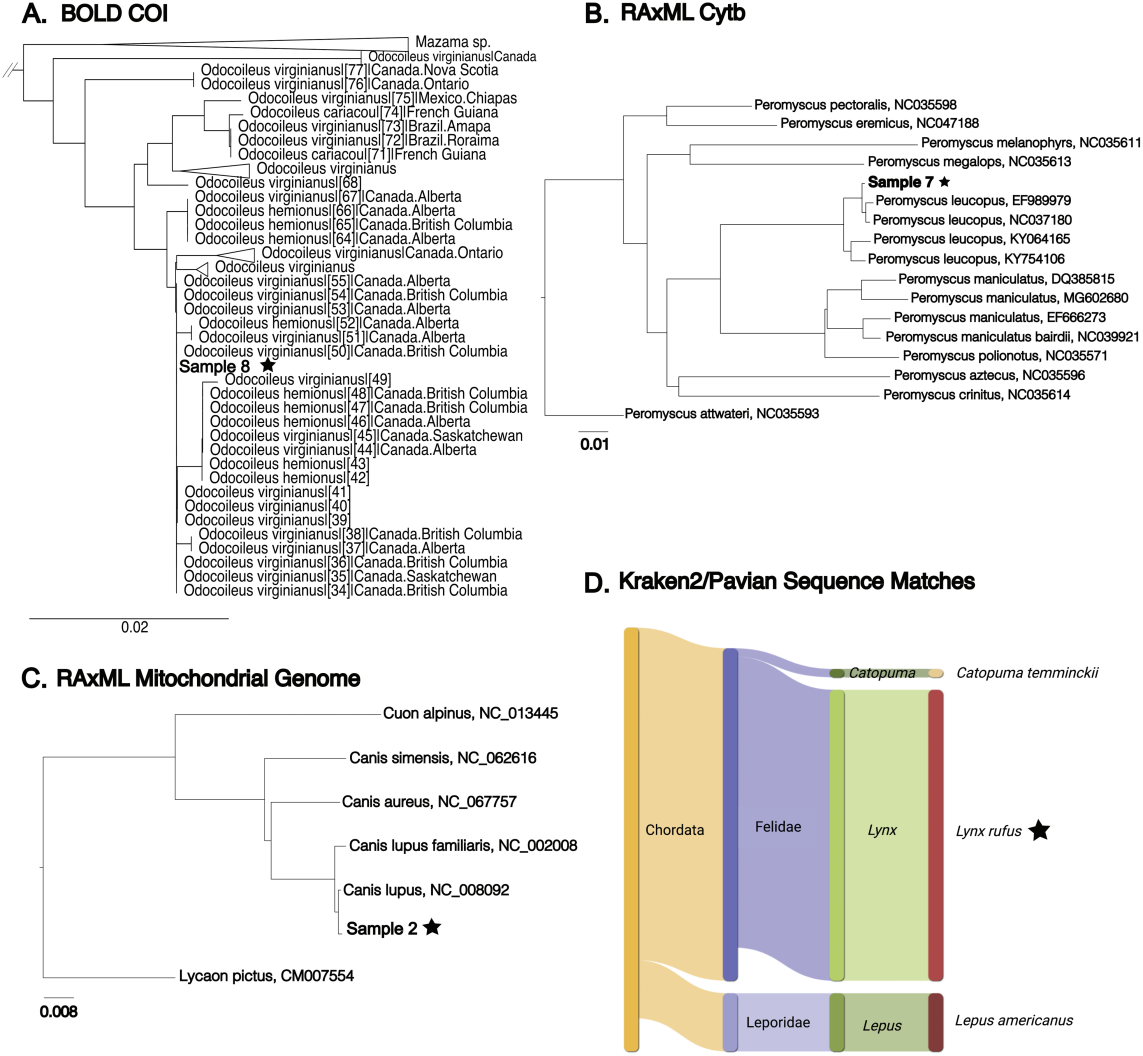
Various bioinformatic methods can be utilized to produce species identification. Samples or the species identification are indicated with the black star. Panel A shows a neighbor-joining phylogenetic tree generated by the BOLD database using *COI* (accession numbers provided in Supplementary Data SD2). Panel B shows a phylogenetic tree generated by RAxML using *Cytb* sequences from Sample 7 and closely related species from NCBI. Panel C shows the full mitochondrial genome of Sample 2 in a RAxML phylogenetic tree with closely related species from NCBI. Panel D shows the Pavian visualization of Kraken 2 database matches. Reads from Sample 5 matched with 3 species, *Catopuma temminckii*, *Lynx rufus*, and *Lepus americanus.* The majority of the reads matched to *Lynx rufus.*

**Fig. 3.**
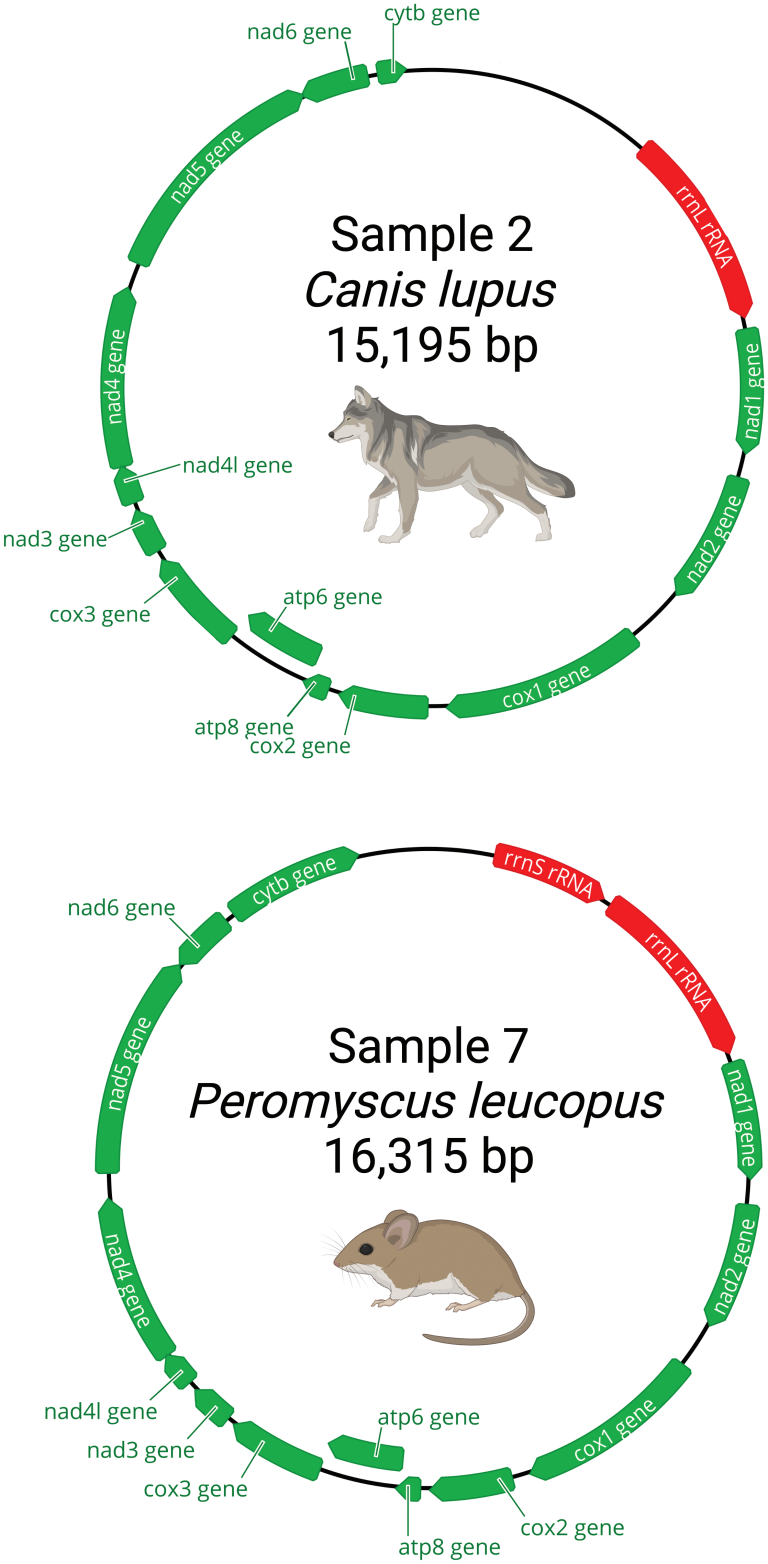
Complete and near-complete mitochondrial genomes from *Peromyscus leucopus* (Sample 7) and *Canis lupus* (Sample 2), respectively. Both mitochondrial genomes were sequenced using NAS. *Canis lupus* mitochondrial genome is near-complete, with approximately 911 bps missing from *Cytb* and small sections of D-loop control region and 12S rRNA. Select annotations include genes and rRNA. Figure created with BioRender.com and Geneious Prime software.

We successfully sequenced mitochondrial sequences and/or particular barcoding genes (*Cytb* and/or *COI*) from all fecal samples using the NAS method, including the near-complete mitochondrial genome of 3 samples. Putative species identifications of blinded samples were initially based on real-time NAS mapping results. Molecular data were generated for each sample through individual sequencing experiments (Table 2). Depending on flow cell type (i.e., Flongle vs. full-sized MinION flow cell), the total number of bases sequenced for each sample ranged from 63,666,885 to 4,909,175,834 bases and total number of reads ranged from 157,425 to 10,502,105 reads. Based on our NAS bioinformatic results, 8 out of 9 of our identifications matched with previous identifications to species level after unblinding sample identifications (Table 2). Output from Kraken 2, Blast, BOLD, and generation of phylogenetic trees produced species identifications (Fig. 2). The BOLD phylogenetic tree that was produced for our Sample 8 showed paraphyletic grouping with 2 closely related species of deer, *Odocoileus virginianus* and *Odocoileus hemionus* (see Discussion). We further investigated the identification of Sample 3 that did not match with the previous PCR identification. Sample 3 was identified by physical characteristics and diameter of the feces as *Canis lupus* (Gray Wolf), then identified by PCR as *Vulpes vulpes* (Red Fox) in Bernstein et al. (2021). All of our bioinformatic analyses for this sample provided support to identify the sample as *C. lupus*—we found no evidence to support the *V. vulpes* identification. From 3 sequencing experiments—Samples 2, 7, and 8—with full-sized flow cells, near-complete mitochondrial genomes were assembled. In Fig. 3, Samples 2 (*C. lupus*) and 7 (*Peromyscus leucopus*) mitochondrial genomes are shown, with 61 and 24X coverage, respectively.

**Table 2.** NAS sequencing experiments for rapid mammalian species barcoding. Reads mapped to the mitogenome database indicate the number of nanopore sequencing reads with matches to the NCBI Refseq database. Total number of mapped bases indicates the total number of bases in the reads that mapped to the NCBI Refseq database.

Sample ID	Flow cell type	Sequencing pores at experiment start	Total bases sequenced	Number of reads	Reads after quality filtering	Reads mapped to mitogenome database	Total bases mapped to mitogenome database
1	Flongle	65	605,520,941	1,311,441	967,924	98	9,644
2	Flow cell	357	4,555,260,593	10,502,105	6,701,295	1,744	14,425
3	Flongle	80	175,525,810	433,311	264,108	41	8,116
4	Flow cell	219	69,091,774	296,158	56,317	47	2,102
5	Flongle	47	273,448,450	615,773	457,829	53	2,845
6	Flow cell	787	4,909,175,834	9,786,381	6,029,765	38	6,173
7	Flow cell	492	2,702,000,834	5,136,945	4,061,666	173	11,024
8	Flow cell	1,490	2,600,199,180	4,755,704	4,320,878	289	618,113
9	Flongle	23	63,666,885	157,425	111,708	24	1,079

## Discussion

We demonstrated that with our NAS workflow, we can successfully identify the species of an excreting host from a sample of their feces. We successfully sequenced sections of mitochondrial DNA, barcoding genes, and/or complete or near-complete mitochondrial genomes of all samples. Eight out of 9 identifications matched original PCR or appearance-based identification and we believe the ninth was originally misidentified by PCR due to a lack of supporting evidence from our study. Most methods for the molecular identification of mammal feces involve a PCR step that is time-consuming and typically requires a brick-and-mortar laboratory setup. Using NAS data combined with the bioinformatic and phylogenetic approaches described in this paper can lead to a rapid (i.e., data generated within hours of sample collection) species identification supported by sequence data and phylogenetic statistics completely free of PCR. Going from fecal sample to species identification quickly is of high value to conservationists and biologists studying difficult-to-find or distinguish species by providing a molecular-based identification in a timely manner. With faster methods for fecal identification, informed management plans or research can be carried out without waiting months for PCR results or visual sightings of species. This study serves as a proof of concept for this method and can be expanded to incorporate the generation of other sequencing data NAS targets.

In line with previous studies focused on mitochondrial-based species identifications, as well as critiques of such methods, extra caution must be used when considering incomplete lineage sorting, mitochondrial capture, and historical or active hybrid zones (Larsen et al. 2010; Thompson et al. 2013; vonHoldt et al. 2016). In certain situations, using NAS for species identification may require extra care, for example, when distinguishing particular wild species and their domestic counterparts (e.g., polecat and domestic ferret, wildcat and domestic cat, wolf and domestic dog). Such examples are hampered by the sharing of mitochondrial DNA haplotypes as a result of incomplete lineage sorting or hybridization (Davison et al. 1999; Randi et al. 2001; Krofel et al. 2022). We found that using multiple bioinformatic tools in combination (i.e., minimap2, Kraken 2, Blast and BOLD search engines, phylogenetic tree generation) could sometimes help to parse out an identification in these situations (i.e., *C. lupus* vs. *C. l. familiaris*). Sample 8 was identified as *O. virginianus* by all bioinformatic methods; however, in the phylogenetic tree generated by BOLD with *COI* sequences, the sample sequence grouped with sequences from both *O. virginianus* and *O. hemionus.* This result coincides with recent evidence supporting that these 2 species underwent a hybridization event approximately 1.32 mya. The resulting mitochondrial capture of the *O. virginianus* mitogenome by *O. hemionus* produced a new haplogroup, along with novel haplogroups produced from introgression during continued hybridization between the 2 species (Wright et al. 2022).

As with all molecular-based species ID analyses of bulk fecal samples, another limitation arises when attempting to differentiate between host and prey species, particularly among carnivores consuming mammalian prey. However, other characteristics of the fecal sample being tested (e.g., physical characteristics, ecological, temporal, etc.) could be used to elucidate host species. For the Kraken 2 analysis of Sample 5, *Lynx rufus* was the match of the majority of reads; however, a small percentage of reads were mapping to *Lepus americanus* and *Catopuma temmnickii*. *Catopuma temmnickii* was ruled out due to only 1 read mapping to this species and geographical range of this species in relation to where our samples were collected. We could rule out a *L. americanus* identification for the depositing host because of the low number of reads mapping to this species compared to the number mapping to *L. rufus.* Physical characteristics of the feces (e.g., total size of the sample) also supported the identification of *L. rufus* over *L. americanus*. However, *L. americanus* are an important prey animal of *L. rufus* (Moen et al. 2012). We note that extracting DNA from mucosal cells found on the exterior of fecal samples would likely enrich the depositing host species DNA and thus would be a useful approach in such situations (Vynne et al. 2012).

While the methods described herein do not require PCR to produce species identifications, PCR amplification could be used to amplify mitochondrial DNA to increase sensitivity and produce more reads at higher coverage, although with the limitations described above. In light of our results, we recommend a minimum depth of coverage for phylogenetically informative mitochondrial genes (e.g., *Cytb* or *COI*) consisting of 40 quality-filtered nanopore reads. We note that this is a minimum for species-level molecular identification—depending on sequencing throughput it is likely that assembly of complete mitochondrial genomes (~16,000 bps) of fecal-host mammals is achievable. With these minimum specifications, it is more likely that a confident identification can be made based on the above methods. A full-sized MinION flow cell (e.g., R10.4) clearly provides greater sequencing depth, thus yielding more mitochondrial host reads for species identification, than does the smaller ONT Flongle. At the time of writing of this paper, the current price for a full-sized flow cell is approximately $900 USD (Oxford Nanopore Technologies 2023). Bulk purchases of flow cells and molecular barcoding of up to 96 samples can reduce cost per sample. Additionally, custom NAS databases can be created to simultaneously target both the mitogenome for identification, as well as other genomic regions of interest from the excreting host or metagenomic community to reduce the need for multiple sequencing experiments. The full-sized flow cells utilized for this project had all been used for other projects and then washed for reuse. Nevertheless, utilizing Flongles significantly reduces total cost (current price $90 USD) for NAS-based molecular barcoding. The Flongles used here all had less than the maximum possible number of pores due to their age. The lowest pore count at the start of a Flongle experiment leading to a successful identification was 24 out of a possible 126 pores. However, given our Flongle data, we recommend a minimum of at least 100 active sequencing pores at the start of an experiment to provide optimal results from individual samples. Even with these reduced pore counts, we still generated enough data to identify our samples, demonstrating another opportunity to reduce cost. We note that continual improvements to ONT sequencing technologies and associated bioinformatics will impact these estimates, lowering cost per sample and increasing sequencing throughput.

The resolving power of NAS-based species identification will increase concordantly with the increase in available mammalian mitochondrial genomes. At the time of this study, no mitochondrial sequences had been published to NCBI for *Sylvilagus floridanus.* Because of this lack of comparative sequences, we made the species identification of Sample 6 by mapping to closely related species and then using metadata including location of sample collection and species with typical ranges in that area. Sample and collection site metadata are an invaluable resource to validate molecularly generated identifications, especially when reference mitochondrial genomes of species of interest are not currently available.

We provide a quick procedure to taxonomically identify mammalian species from a fecal sample. With our nanopore sequencing workflow, from the time a fecal sample is acquired to bioinformatic analysis of DNA sequence data, a species identification can be achieved in less than 12 h, depending on the desired amount of data. In our study, we ran sequencing experiments up to 48 h; however, a putative identification can be achieved through NAS alignment of reads and/or basic bioinformatic analysis within minutes to hours of starting the sequencing experiment. For this reason, we believe that the entire workflow can be completed in less than 12 h, in some cases. The adaptive sampling method allows for fecal mitochondrial DNA to be enriched and targeted for sequencing, which has previously required a PCR step. There is potential to use NAS to generate entire reference quality mitochondrial genomes while documenting microbiomes and diet components such as consumed prey species and vegetation. Conducting in-the-field extraction and sequencing of DNA with a mobile lab, facilitated by the portability of the ONT MinION and miniaturization of required lab equipment, could provide even more flexibility and speed for molecular species identifications on site.

Future studies could apply this framework to detect barcoding genes and whole genomes of fecal pathogens. We anticipate that NAS of fecal samples for species identification can have broad utility for applications where rapid species identifications are needed including conservation, biodiversity studies, invasive species detection, illegal wildlife trade, marine mammal studies, and forensic studies that require fast and accurate species identification (Iyengar 2014; Morisette et al. 2020; Henger et al. 2023). Many North American Indian tribes are in the process of inventory and monitoring of biodiversity on tribal and ceded lands. The northeastern Ojibwe tribes acknowledged in this study are all leading initiatives to measure biodiversity and map and document the spread of invasive species as climate adaptation plans are implemented (Moore et al. 2014; Stults et al. 2016). In light of the results presented herein, alongside continued improvement in nanopore sequencing chemistries and bioinformatic pipelines, we anticipate targeted NAS-based species barcoding to gain broad acceptance by the scientific community (Franco-Sierra and Díaz-Nieto 2020). Such methods will likely usher in an exciting new era of species monitoring and discovery to the field of mammalogy.

## Supplementary data

Supplementary data are available at *Journal of Mammalogy* online.

**Supplementary Data SD1.**—Detailed bioinformatic workflow including, software packages and versions, commands used for species identification, and explanation of usage.

**Supplementary Data SD2.***—*Metadata from the neighbor-joining phylogenetic tree generated by the BOLD database using the *COI* sequence from Sample 8, identified as *Odocoileus virginianus*.

gyae044_suppl_Supplementary_Data_1

gyae044_suppl_Supplementary_Data_2

## Data Availability

All sequences associated with this project are uploaded to the SRA Database with the Project ID: PRJNA1026962.
